# Optimization of Medium Composition and Fluidized Bed Drying Conditions for Efficient Production of Dry Yeast

**DOI:** 10.3390/microorganisms13010022

**Published:** 2024-12-26

**Authors:** Hyun-Jin Kang, Hwan Hee Yu, Chang-Won Cho, Young Kyung Rhee, Tae-Wan Kim, Young-Wook Chin

**Affiliations:** 1 Research Group of Traditional Food, Korea Food Research Institute, Iseo-myeon, Wanju-gun 55365, Jeollabuk-do, Republic of Korea; 2 Food Standard Research Center, Korea Food Research Institute, Iseo-myeon, Wanju-gun 55365, Jeollabuk-do, Republic of Korea

**Keywords:** dry yeast, fluidized bed drying, medium composition, response surface methodology, batch fermentation

## Abstract

Yeast formulations such as dry yeast are essential for supplying microbial starters to the alcoholic beverage industry. In Korea, the expensive freeze-drying method is used to manufacture brewer’s dry yeast, and therefore an economical process such as fluidized bed drying is needed. In the dry yeast manufacturing process, the medium and drying conditions are key factors that determine its quality and manufacturing cost. In this study, we aimed to optimize the medium composition and fluidized bed drying conditions for the efficient production of dry yeast. Muscovado and corn steep liquor were used as the carbon and nitrogen sources, respectively, and their optimal concentrations were identified using response surface methodology for efficient cultivation of *Saccharomyces cerevisiae* ReY4-7 isolated from *nuruk*. Central composite design analysis revealed that the optimal medium composition was 146.12 g/L muscovado and 58.68 g/L corn steep liquor. A dry cell weight of 36 g/L was achieved during 24 h of batch fermentation in a 30-L bioreactor containing this medium. Analysis of protective agents against fluidized bed drying revealed Span 80 as the strongest protective agent for *S. cerevisiae* ReY4-7. Response surface methodology revealed 50 °C and 41.45 min as the optimal fluidized bed drying conditions, under which the viable cell count reached 10.28 log CFU/g, comparable to that of the commercial dry yeast products. Overall, optimization of the medium and drying conditions significantly improved the final cell concentration in the cultivation process and the viable cell count in the drying process of dry yeast.

## 1. Introduction

Yeast produces ethanol as well as various higher alcohols, esters, and organic acids from raw materials, such as rice, acting as a major factor determining the quality of alcoholic beverages [1]. In Korea, only some alcoholic beverage manufacturers cultivate yeast, whereas most companies mainly import yeast formulated as granular powder, which is easy to store and distribute, for wine and bakery products. Recently, the isolation of Korean indigenous yeast with excellent brewing characteristics has increased, further increasing its application for Korean alcoholic beverage production [1,2,3,4,5]. The Korean indigenous yeasts are mainly isolated from *nuruk*, which is a traditional Korean brewing starter [1,2,3,4,5]. Since *nuruk* is made by naturally fermenting grains such as rice, it has a very diverse microbial population [6].

Currently, all Korean dry yeasts in the market are manufactured using the freeze-drying method, which first freezes the cells and then sublimates the ice under reduced pressure. The freeze-drying method has a longer drying time and much higher manufacturing cost than other drying methods, making it unsuitable for brewing-yeast manufacture. Comparatively, fluidized bed drying is more suitable for the cultivation of dry yeast for alcoholic beverage production as the raw material is floated in a hot air stream and dried, resulting in fast and uniform heat transfer and low manufacturing cost. However, due to the use of high-temperature air, the optimization of the drying temperature and time and the selection of appropriate protective agents are essential to maintain the yeast viability in this method. Several studies have demonstrated the efficacy of fluidized bed drying in preserving the viability and quality of yeast cells during drying. Vorländer et al. (2023) revealed that the drying temperature and protective agent play important roles in maintaining cell viability, with a 45–50 °C drying temperature and appropriate protective agents, such as glycerol, significantly improving the cell recovery and function after drying [7]. Mille et al. (2005) reported that fluidized bed drying facilitates the uniform heat distribution and rapid moisture removal essential for large-scale industrial applications and that emulsifiers, such as Span 80, stabilize yeast membranes at high temperatures [8].

The medium is another important factor for the industrial fermentation process, accounting for 25% to 70% of the total cost [9]. An ideal medium allows the cells to rapidly proliferate in a given volume. The fed-batch method is commonly used for high-density cell culture; however, it poses a higher risk of contamination than batch culture, with a more complex and difficult-to-scale-up process because the feeding rate of additional nutrients must be continuously changed depending on the cell growth. Notably, the batch and fed-batch methods result in similar viable cell counts [10]. Therefore, in the industrial cultivation process of brewing yeast starter, it is desirable to obtain high cell concentration by culturing in batch mode while using inexpensive medium components.

Many studies, including those on the optimization of medium components using the response surface methodology (RSM), have investigated high-density yeast cell cultures. In labs, expensive materials, such as yeast extract, amino acids, and vitamins, are used to produce high-value-added metabolites as final products [11]. However, for industrial-scale production of cheap products, such as yeast cells and bioethanol, cheap and renewable materials, such as molasses and corn steep liquor (CSL), are mainly used owing to their economic feasibility.

Molasses, a by-product of sugar processing, is composed of water (17–25%), sugar (39–61%), nitrogenous compounds (2–6%), vitamins (pyridoxine, thiamine, riboflavin, folic acid, biotin, and pantothenic acid), and trace elements [12,13]. Muscovado (MS) is non-centrifuged (unrefined) cane sugar containing natural molasses. Although MS is generally more expensive than molasses because it is produced organically in small quantities, its cost can vary greatly depending on the manufacturing process and quality; it has approximately twice the sugar (sucrose, glucose, and fructose) content of molasses, making it a viable option as a carbon source for yeast. CSL, a by-product of corn wet milling, is among the cheapest nitrogen sources [14]. It contains proteins (30–50%), amino acids, minerals, and vitamins, serving as a nutrient replacement for expensive complex media, such as yeast extract and peptone [15,16,17]. Use of CSL reduces the cost of lipase production by 55–60% [15]. Moreover, CSL effectively replaces the yeast extract in the fermentation of hydrolyzed waste starch stream to produce ethanol, without changing the reaction kinetics [18].

In our previous study, *Saccharomyces cerevisiae* ReY4-7 was isolated from *nuruk* [19]. The ReY4-7 strain showed excellent ability to produce ethanol as well as flavor compounds including various esters and higher alcohols [19]. Since it is a promising strain that could be commercialized in the Korean brewing industry, ReY4-7 was employed as a model strain in this study. We aimed to optimize the production process for dry yeast by (1) optimizing the medium composition to maximize the final cell concentration in the yeast cultivation process and (2) optimizing the emulsifier, drying temperature, and time in the fluidized bed drying process to maximize the viable cell count of dry yeast.

## 2. Materials and Methods

### 2.1. Yeast Strain and Culture Conditions

*S. cerevisiae* ReY4-7 (KFCC11964P) previously isolated from *nuruk* (traditional Korean starter for brewing) was used for dry yeast production [19]. Pre-culture of ReY4-7 strain was performed in a test tube containing 5 mL of yeast peptone dextrose (YPD; Difco, Detroit, MI, USA) or a flask containing 100 mL of YPD at 30 °C and 220 rpm for 18 h. MS (Raw Brown Sugar Milling Co., Pamplona, Philippines), CSL (Aladdin, Shanghai, China), and trace element solution (TES; (NH_4_)_2_(SO_4_), KH_2_PO_4_, MgSO_4_, ZnSO_4_, and biotin) were used for medium optimization experiments. The optimized medium was designated as MC medium (146.12 g/L MS and 58.68 g/L CSL). Pilot-scale cultivation was performed in a 30-L bioreactor containing 15 L of MC medium at 30 °C, 120 rpm, and 0.1 vvm for 24 h. YPD, potato dextrose broth (PDB), YED (142.5 g/L glucose, 10 g/L yeast extract, 5 g/L KH_2_PO_4_, and 1 g/L MgSO_4_), WSC (142.5 g/L white sugar and 58.6 g/L CSL), BSC (142.5 g/L black sugar and 58.6 g/L CSL), and MC2 (146.1 g/L molasses and 58.6 g/L CSL) media were compared, and ReY4-7 strain was cultivated in a flask containing 50 mL of the prepared media at 30 °C and 220 rpm for 24 h.

### 2.2. Optimization of Medium Composition for Improving Final Cell Concentration Using RSM

RSM was used to optimize the medium composition, and the experiments were designed using a central composite design (CCD). Experimental conditions were set with MS (g/L, X_1_) and CSL (g/L, X_2_) as independent variables, and experimental range was coded into five levels (–1.414, –1, 0, 1, and 1.414) according to the central composite circumscribed (CCC) model. The pre-cultured cells were inoculated at 1% (*v*/*v*) into the 11 run conditions, including three center points, as per the CCC design. The cultures were incubated at 30 °C and 220 rpm for 24 h, and dry cell weight (DCW, g/L) was used as the dependent variable.

### 2.3. Fluidized Bed Drying

To measure the viable cell count of dry yeast with various emulsifiers, experiments were conducted using a fluidized bed dryer (Sherwood Scientific, Cambridge, UK). *S. cerevisiae* ReY4-7 was pre-cultured, as described above, and 10 mL of the pre-culture was inoculated into 1 L of YPD and cultured under the same conditions. Based on the OD_600 nm_ value of the main culture, various emulsifiers (Span 20, Span 60, Span 80, and Span 83) were added at 1% (*w*/*w*) relative to the dry cell mass of *S. cerevisiae* ReY4-7, followed by stirring. The mixture was centrifuged at 2800× *g* for 20 min to collect the cells. After removing moisture from the collected cells, fluidized bed drying was performed under the following conditions: inlet temperature of 50 °C, drying time of 30 min, flow rate of 33.33 L/s, bed temperature of 44.5 °C, and bed relative humidity of 18.7%.

### 2.4. Measurement of Cell Concentration, Viable Cell Count, and Moisture Content

Dry cell weight (DCW) was determined using optical density and a conversion factor (0.296). Optical density was measured at 600 nm absorbance using a spectrophotometer (Eppendorf Biospectrometer, Hamburg, Germany) after the samples were diluted to keep optical density between 0.1 and 0.5. To determine the conversion factor, 1 mL of cell suspension was filtered through pre-weighed filter papers. The filter paper was dried for 15 min in a microwave, cooled down in a desiccator, and weighed [20].

To measure viable cell count, after adding 9 mL of sterile water to 1 g of dry yeast, serial dilutions were performed, and the diluted solution was inoculated onto YPD plates (10 g/L yeast extract, 20 g/L Bacto peptone, 20 g/L glucose, and 15 g/L agar). The plates were incubated at 30 °C for 24 h, and the number of yeast colonies formed was counted and expressed as log CFU/g of dry yeast. Then, 3 g of dry yeast was placed on a weighing dish, and the moisture content was measured using a moisture analyzer (MB120; Ohaus Corporation, Waukegan, IL, USA).

### 2.5. Optimization of Fluidized Bed Drying Conditions Using RSM

RSM was used to optimize the yeast drying conditions via fluidized bed drying, and the experiment was designed according to CCD. The experimental conditions were set, with the drying temperature (°C; X_3_) and drying time (min; X_4_) as independent variables coded at three levels (–1, 0, and 1) based on the central composite face-centered design. The experiment was conducted over 11 runs, including three center points. Viable cell count (log CFU/g; Y_2_) and moisture content (%; Y_3_) were the dependent variables. The ReY4-7 strain cultured under the conditions mentioned above in a 30-L bioreactor was used to optimize drying conditions.

### 2.6. Statistical Analysis and Response Surface Model Validation

Each experiment was repeated three times, and the results are represented as the mean ± standard deviation. All results were analyzed using the SPSS package (v.12.0K; SPSS Inc., Chicago, IL, USA). Significant differences among samples were analyzed via one-way analysis of variance (ANOVA), with significance set at *p* < 0.05, and homogeneous groups were classified via Duncan’s multiple-range test. Based on the CCD experimental results, the significance and fit of the response surface model were verified via ANOVA and regression analysis of each independent and dependent variable. Characteristics of the variables and corresponding optimal conditions were determined using the Design-Expert software (v.11.1.2.0; Stat-Ease, Inc., Minneapolis, MN, USA). The predictive regression equation for the dependent variable (Y) in relation to the independent variables (X_1_, X_2_) was expressed as follows:

Y = β_0_ + β_X1_X_1_ + β_X2_X_2_ + β_X1X2_X_1_X_2_ + β_X1²_X_1_^2^ + β_X2²_X_2_^2^ (1)

β_0_, constant coefficient
β_i_, linear coefficient (i = X_1_, X_2_)
β_ii_, second order coefficient (ii = X_1_^2^, X_2_^2^)

## 3. Results and Discussion

### 3.1. Optimization of Medium Composition for Improving Final Cell Concentration of S. cerevisiae ReY4-7

As a preliminary study, three-factor RSM was conducted using MS, CSL, and TES as independent variables. Unlike MS and CSL, TES did not significantly affect the growth of *S. cerevisiae* ReY4-7 (Tables S1 and S2), possibly because MS and CSL contain sufficient amounts of essential nutrients and minerals. Specifically, CSL contains nitrogen, vitamins, and minerals necessary for yeast growth [21]. Excessive addition of trace elements, such as zinc, copper, and manganese, inhibits yeast cell division and enzyme activity, thereby negatively impacting cell growth [22]. Therefore, in this study, optimization was conducted using only MS and CSL.

To maximize the final cell concentration of *S. cerevisiae* ReY4-7, RSM was performed with independent variables and their ranges set according to CCC. The medium composition and corresponding DCW for the 11 intervals designed based on CCC are presented in Table 1.

The regression Equation (2) of the dependent variable, DCW (Y_1_), was derived through ANOVA and regression analysis, and the model fit of the dependent variable was evaluated using the following equation:

Y_1_ = 31.47 + 1.45X_1_ + 1.56X_2_ − 1.92X_1_X_2_ − 2.38X_1_^2^ − 4.93X_2_^2^ (2)

ANOVA revealed that the *p*-values of the model for DCW were below 0.0001, indicating a high level of significance, and that the R^2^ value of the regression equation was 0.9907, showing a high reliability (>0.95; Table 2). In contrast, the *p*-value of the lack of fit was 0.1599, which was above 0.05, indicating no significant difference. This confirmed that the response surface model was suitable to explain the changes in the DCW of the cells according to the medium composition.

**Figure 1:**
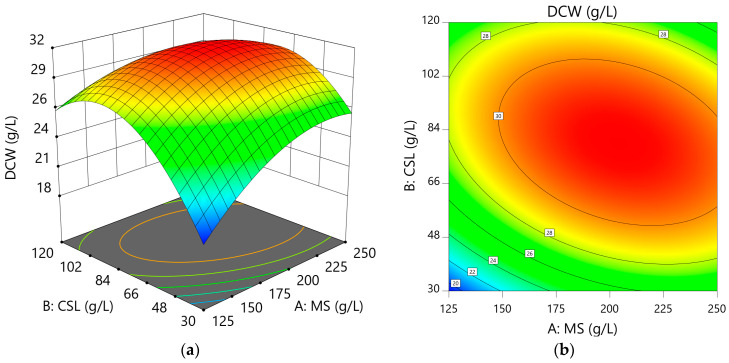
Three-dimensional (3D) response surface (**a**) and contour plot (**b**) of the effects of muscovado (MS; X_1_) and corn steep liquor (CSL; X_2_) on final dry cell weight (DCW).

Using the regression spinning Equation (2), changes in DCW according to the concentrations of MS and CSL are shown in Figure 1. Next, the optimal medium composition to maximize the cell concentration and minimize the MS and CSL concentrations to ensure economic feasibility was predicted. The predicted optimal medium composition was 146.12 g/L MS and 58.68 g/L CSL, with a predicted DCW of 27.80 g/L.

To verify the predictive accuracy of the response surface model, flask culture was performed using the optimal medium composition. The DCW under the optimal medium was 27.6 g/L. Compared to the predicted value, the experimental value did not show a significant difference at the 95% confidence level (27–29 g/L), confirming the suitability of the response surface model. The optimized medium was designated as the MC medium.

**Figure 2:**
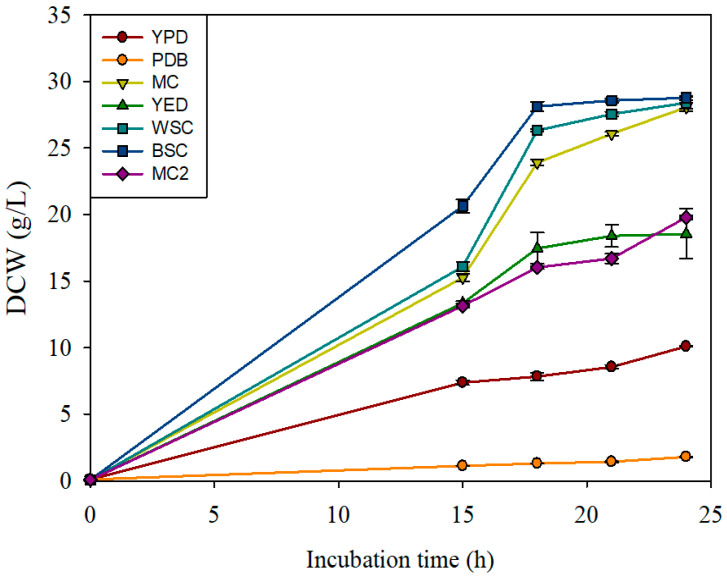
Comparison of the growth profiles of *Saccharomyces cerevisiae* ReY4-7 in different media. Values are represented as the mean ± standard deviation (SD; n = 3). YPD, yeast peptone dextrose; PDB, potato dextrose broth; MC, MS + CSL (optimized medium); YED, glucose + yeast extract + KH_2_PO_4_ + MgSO_4_; WSC, white sugar + CSL; BSC, black sugar + CSL; MC2, molasses + CSL.

To assess the performance of the MC medium, cell growth was compared to that in other media (Figure 2). The MC medium showed the third fastest cell growth rate following the WSC and BSC media containing white and brown sugar, respectively. The final DCW after 24 h of culture was 28.1 g/L in the MC medium, which was similar to the values in the WSC and BSC media (28.4 g/L and 28.8 g/L, respectively). Notably, the final DCW in the MC medium was 60% higher than that in the MC2 medium containing molasses as the carbon source. Compared to those in the YED, YPD, and PDB complex media, the final DCW values in the MC medium were 1.5, 2.8, and 15.7 times higher, respectively. These results confirmed that MS can be used as a carbon source for yeast. Finally, batch fermentation was performed in a 30-L bioreactor for better growth conditions, including aeration. After 24 h of culture, the DCW was 35.5 g/L, which was approximately 30% higher than that in the flask culture (Figure S1).

### 3.2. Effects of Emulsifier on Viable Cell Count After Fluidized Bed Drying

**Figure 3:**
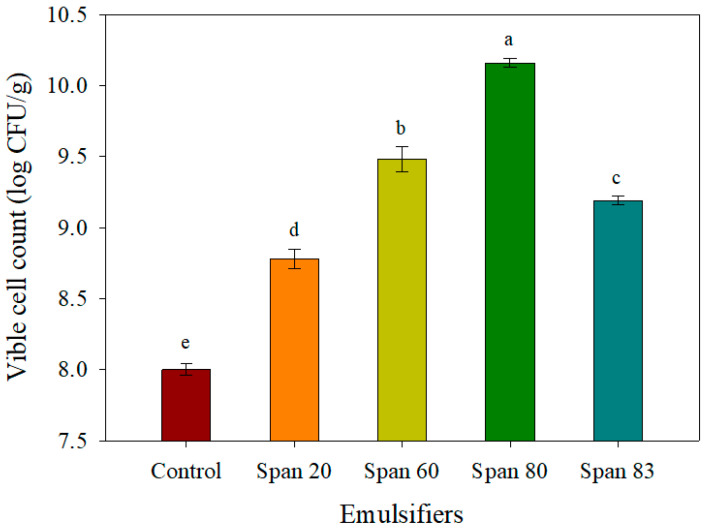
Effect of emulsifier type on the viable cell count after fluidized bed drying. No emulsifier was added in the control. Values are represented as the mean ± SD (n = 3). Different small letters (a–e) on the bar indicate the significant differences determined via Duncan’s multiple-range test (*p* < 0.05).

Span emulsifier is widely used as a protective and rehydration agent for active dry yeast. There are some kinds of Span including Span20, 60, 80, and 83 according to its hydrophilic–lipophilic balance (HLB). Although Span 60 is mainly used for active dry yeast, its protective effects may be dependent on yeast strain. Thus, the viable cell count of the fluidized-bed-dried yeast according to the type of emulsifier was investigated to search for an optimal Span emulsifier for the ReY4-7 strain (Figure 3). The results showed that Span 80, followed by Span 60, Span 83, and Span 20, exhibited the highest yeast viable cell count (10.16 ± 0.03 log CFU/g) in the experimental group.

The addition of 1–2% of emulsifiers with HLB values of 4–7 has been reported to be very effective in reducing the loss of cell components during rehydration and mechanical disruption of dry yeast [23]. The HLB values of Span 20, Span 60, Span 80, and Span 83 are 8.6, 4.7, 4.3, and 3.7, respectively. Thus, Span 80 and Span 60 formed a protective film around the cell membrane and reduced water loss by stabilizing the water/oil emulsions owing to their low HLB values (lipophilicity), whereas Span 20 formed oil/water emulsions owing to its high HLB values (hydrophilicity), thereby accelerating water loss from the cell membrane. On the other hand, Span 83, which has the lowest HLB value, showed a lower protective effect on yeast cells compared to Span 60 and Span 80. Although Span 83 (HLB 3.7) is slightly more lipophilic than Span 80 (HLB 4.3), its relatively strong lipophilicity weakens the interaction with the yeast cell membrane, which also reduces the yeast cell protection effect [7]. Therefore, the strong protective effects of lipophilic emulsifiers are possibly due to their ability to bind to the cell membrane to form a protective layer [24,25].

### 3.3. Effects of Drying Temperature and Time of Fluidized Bed Drying on the Viable Cell Count and Moisture Content of Dry Yeast

**Figure 4:**
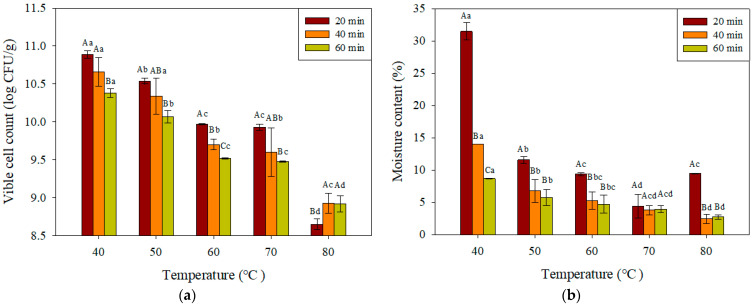
Effects of drying temperature and time of fluidized bed drying on the viable cell count (**a**) and moisture content (**b**) of dry yeast. Values are represented as the mean ± SD (n = 3). Different capital letters (A–C) and small letters (a–d) on the bar indicate the significant differences based on the drying time and temperature determined via Duncan’s multiple-range test (*p* < 0.05).

Changes in viable cell count and moisture content were evaluated at different fluidized bed drying temperatures (40, 50, 60, 70, and 80 °C) and time points (20, 40, and 60 min) (Figure 4). Both the viable cell count and moisture content decreased with increasing drying temperature. High-temperature drying denatures ATPase, which maintains the pH in yeast, disrupting redox regulation in the cytoplasm and promoting reactive oxygen species generation during respiration, thereby causing severe oxidative damage to cells [7,26,27]. Unlike at other temperatures, at 80 °C, viable cell counts were higher at the drying times of 40 and 60 min than at 20 min. The survival rate of yeast increases as the drying time increases at 80 °C, possibly due to the formation of a crust on the cell surface via heat curing at high temperatures, which reduces heat transfer and thermal conductivity, thereby inhibiting moisture evaporation [28,29].

### 3.4. Optimization of Fluidized Bed Drying Conditions Using RSM

To optimize the fluidized bed drying conditions, RSM was performed by setting the independent variables and their ranges according to CCD. The drying conditions for the 11 runs designed according to CCD and the results of the dependent variable, viable cell count, and moisture content analyses are presented in Table 3. Regression Equations (3) and (4) for the dependent variables, viable cell count (log CFU/g; Y_2_), and moisture content (%; Y_3_), derived via ANOVA and regression analysis, are as follows:

Y_2_ = 9.70 − 0.32X_3_ − 0.23X_4_ + 0.01X_3_X_4_ + 0.26X_3_^2^ + 0.04X_4_^2^ (3)

Y_3_ = 5.36 − 1.53X_3_ − 2.15X_4_ + 1.07X_1_X_2_ − 0.23X_3_^2^ + 1.46X_4_^2^ (4)

These equations were used to evaluate the suitability of the model for the dependent variables.

ANOVA revealed that the *p*-values of the models for viable cell count and moisture content were below 0.0001, indicating a high level of significance, and that the R^2^ values for the regression equations were 0.9869 and 0.9969, respectively, showing high reliability (>0.95; Table 4 and Table 5). Moreover, the *p*-values for the lack of fit were 0.7161 and 0.0662 for the viable cell count and moisture content, respectively, with both above 0.05, indicating no significant difference. These results confirmed that the response surface model was suitable to explain the effects of the drying temperature and time on the viable cell count and moisture content of yeast.

**Figure 5:**
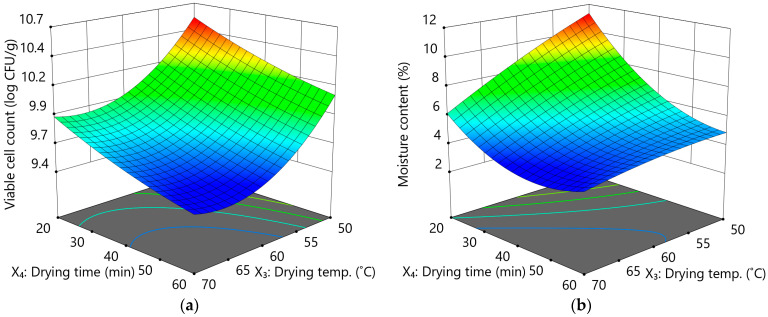
3D response surface models of the effects of drying time and temperature on the viable cell count (**a**) and moisture content (**b**) of yeast.

Using regression Equations (3) and (4), changes in the viable cell count and moisture content according to the drying temperature and time are shown in Figure 5. To optimize the fluidized bed drying conditions, drying conditions to achieve the highest viable cell count and moisture content < 10% were predicted. The predicted optimum drying temperature and time were 50 °C and 41.45 min, respectively, and the viable cell count and moisture content were 10.27 log CFU/g and 6.42%, respectively. Subsequently, fluidized bed drying experiments were conducted under the optimal conditions to verify the predictive accuracy of RSM. Viable cell count and moisture content were 10.28 log CFU/g and 6.65%, respectively. Compared to the predicted values, experimental values did not show significant differences at the 95% confidence level (10.14–10.40 log CFU/g and 6.00–6.85%, respectively), confirming the suitability of the response surface model. Sullivan and Bradford (2011) reported the viable cell counts of five commercial active dry yeast products as 8–13 log CFU/g. In this study, viable cell count was 10.28 log CFU/g under optimal conditions, which is higher than or similar to those of commercial products [30].

## 4. Conclusions

In this study, we assessed the optimal medium composition and drying conditions for the efficient production of dry yeast using fluidized bed systems. Medium optimization was performed using RSM, and optimal concentrations of MS and CSL were determined to maximize the cell concentration. As a protective agent, Span 80 exerted strong protective effects during drying. Additionally, the optimal fluidized bed drying conditions were determined as 50 °C and 41.45 min, which resulted in viable cell counts similar to or higher than those of commercial dry yeast. Overall, our results can aid in the effective production of dry yeast with high cell viability and broad applications in the food and fermentation industries.

## Figures and Tables

**Table 1:** Central composite design (CCD) for medium optimization using muscovado (MS) and corn steep liquor (CSL) and final dry cell weight (DCW) for *Saccharomyces cerevisiae* ReY4-7.

Run	Independent Variable		Dependent Variable
	Muscovado (g/L) (X_1_)	Corn Steep Liquor (g/L) (X_2_)	DCW (g/L) (Y_1_)
1	125	30	19.60
2	250	30	26.04
3	125	120	25.80
4	250	120	24.55
5	99.11	75	24.60
6	275.88	75	29.16
7	187.5	11.3	19.02
8	187.5	138.64	24.54
9	187.5	75	31.13
10	187.5	75	31.75
11	187.5	75	31.53

**Table 2:** Analysis of variance (ANOVA) of the response surface quadratic model for medium optimization using MS and CSL.

Variables	Sum of Square	DF ^(1)^	Mean Square	F-Value ^(2)^	*p*-Value ^(3)^
Model	193.73	5.00	38.75	106.83	<0.0001
X_1_	16.90	1.00	16.90	46.59	0.0010
X_2_	19.55	1.00	19.55	53.90	0.0007
X_1_X_2_	14.79	1.00	14.79	40.76	0.0014
X_1_^2^	31.99	1.00	31.99	88.21	0.0002
X_2_^2^	137.15	1.00	137.15	378.12	<0.0001
Residual	1.81	5.00	0.36		
Lack of fit	1.61	3.00	0.54	5.41	0.1599
Pure error	0.20	2.00	0.10		
Cor total	195.55	10.00			
		Std. dev.	0.60	R ^(2 4)^	0.9907
		Mean	26.16	Adj-R^2^	0.9815
		CV%	2.30	Pre-R^2^	0.9390
				Adeq Pre	27.5353

^(1)^ Degrees of freedom. ^(2)^ Fisher test. ^(3)^ Significance set at *p* < 0.05. ^(4)^ Coefficient of determination.

**Table 3:** CCD for the optimization of fluidized bed drying conditions, viable cell count, and moisture content of dried yeast.

Run	Independent Variable		Dependent Variable	
	Drying Temperature (°C) (X_3_)	Drying Time (min) (X_4_)	Viable Cell Count (log CFU/g) (Y_2_)	Moisture Content (%) (Y_3_)
1	70	60	9.48	3.93
2	50	60	10.07	4.87
3	60	60	9.52	4.74
4	60	40	9.65	5.21
5	60	20	9.97	9.19
6	70	20	9.93	6.01
7	60	40	9.68	5.32
8	50	40	10.34	6.78
9	60	40	9.78	5.25
10	50	20	10.54	11.23
11	70	40	9.60	3.76

**Table 4:** ANOVA of viable cell count in the response surface quadratic model for the optimization of fluidized bed drying conditions.

Variables	Sum of Square	DF ^(1)^	Mean Square	F-Value ^(2)^	*p*-Value ^(3)^
Model	1.14	5	0.23	75.45	0.0001
X_3_	0.63	1	0.63	207.77	<0.0001
X_4_	0.31	1	0.31	101.71	0.0002
X_3_X_4_	0.00	1	0.00	0.04	0.8488
X_3_^2^	0.17	1	0.17	56.36	0.0007
X_4_^2^	0.00	1	0.00	1.55	0.2685
Residual	0.02	5	0.00		
Lack of fit	0.01	3	0.00	0.51	0.7161
Pure error	0.01	2	0.00		
Cor total	1.16	10			
		Std. dev.	0.06	R ^(2 4)^	0.9869
		Mean	9.87	Adj-R^2^	0.9738
		CV%	0.56	Pre-R^2^	0.9264
				Adeq Pre	27.0851

^(1)^ Degrees of freedom. ^(2)^ Fisher test. ^(3)^ Significance set at *p* < 0.05. ^(4)^ Coefficient of determination.

**Table 5:** ANOVA of moisture content in the response surface quadratic model for the optimization of fluidized bed drying conditions.

Variables	Sum of Square	DF ^(1)^	Mean Square	F-Value ^(2)^	*p*-Value ^(3)^
Model	51.78	5	10.36	322.91	<0.0001
X_3_	14.08	1	14.08	438.96	<0.0001
X_4_	27.68	1	27.68	863.08	<0.0001
X_3_X_4_	4.56	1	4.56	142.17	0.0001
X_3_^2^	0.14	1	0.14	4.34	0.0915
X_4_^2^	5.40	1	5.40	168.31	<0.0001
Residual	0.16	5	0.03		
Lack of fit	0.15	3	0.05	14.27	0.0662
Pure error	0.01	2	0.00		
Cor total	51.94	10			
		Std. dev.	0.18	R ^(2 4)^	0.9969
		Mean	6.03	Adj-R^2^	0.9938
		CV%	2.97	Pre-R^2^	0.9785
				Adeq Pre	58.5109

^(1)^ Degrees of freedom. ^(2)^ Fisher test. ^(3)^ Significance set at *p* < 0.05. ^(4)^ Coefficient of determination.

## Data Availability

The original contributions presented in this study are included in the article/Supplementary Material. Further inquiries can be directed to the corresponding author.

## Supplementary Materials

The following supporting information can be downloaded at: https://www.mdpi.com/article/10.3390/microorganisms13010022/s1, Figure S1: growth profile of *S. cerevisiae* ReY4-7 in the 30 L-bioreactor containing MC medium; Table S1: CCD for medium optimization using MS, CSL, and TES and the results of final dry cell weight (DCW) of *S. cerevisiae* ReY4-7; Table S2: the ANOVA for response surface quadratic model of the medium optimization using MS, CSL, and TES.
