# Supplementary material for: Optimization of Medium Composition and Fluidized Bed Drying Conditions for Efficient Production of Dry Yeast
Source: Microorganisms. 2024 Dec 26;13(1):22. doi: 10.3390/microorganisms13010022 (PMC11767331; doi:10.3390/microorganisms13010022)

Supplementary data

Table S1. CCD for medium optimization using MS, CSL, and TES and the results of final dry cell weight (DCW) of *S. cerevisiae* ReY4-7

Run	Independent variable			Dependent variable
	Muscovado (g/L) (X_1)	Corn steep liquor (g/L) (X_2)	TES (mL/L) (X_3),	DCW (g/L) (Y_1)
1	10	10	0.25	6.00
2	30	10	0.25	7.89
3	10	30	0.25	8.41
4	30	30	0.25	15.21
5	10	10	0.75	6.18
6	30	10	0.75	8.04
7	10	30	0.75	9.11
8	30	30	0.75	14.25
9	3.18	20	0.50	4.45
10	36.82	20	0.50	10.91
11	20	3.18	0.50	3.92
12	20	36.82	0.08	10.46
13	20	20	0.92	9.78
14	20	20	0.50	10.36
15	20	20	0.50	9.44
16	20	20	0.50	10.17
17	20	20	0.50	9.75
18	20	20	0.50	9.50

Table S2. The ANOVA for response surface quadratic model of the medium optimization using MS, CSL, and TES

Variables	Sum of square	DF[1)]	Mean square	F-value[2)]	*p*-value[3)]
Model	133.23	9	14.80	12.74	0.0008
X_1	51.63	1	51.63	44.43	0.0002
X_2	0.09	1	0.09	0.08	0.7847
X_3	36.92	1	36.92	31.77	0.0005
$X_1 X_2$	0.33	1	0.33	0.29	0.6075
$X_1 X_3$	25.96	1	25.96	22.33	0.0015
$X_2 X_3$	4.73	1	4.73	4.07	0.0784
X_1^2	3.33	1	3.33	2.87	0.1289
X_2^2	1.06	1	1.06	0.91	0.3678
X_3^2	8.34	1	8.34	7.18	0.0280
Residual	9.30	8	1.16		
Lack of Fit	8.85	5	1.77	11.86	0.0344
Pure error	0.45	3	0.15		
Cor total	142.53	17			
Std. dev.	1.08		R^2 [4)]	0.9348	
Mean	9.10		Adj-R^2	0.8614	
CV %	11.84		Pre-R^2	0.4965	
			Adeq Pre	12.8258	

[1)]Degrees of freedom

[2)]Fisher test

[3)]Significant at $p<0.05$ and not significant $p>0.05$

[4)]Coefficient of determination

Figure S1. Growth profile of *S. cerevisiae* ReY4-7 in the 30 L-bioreactor containing MC medium

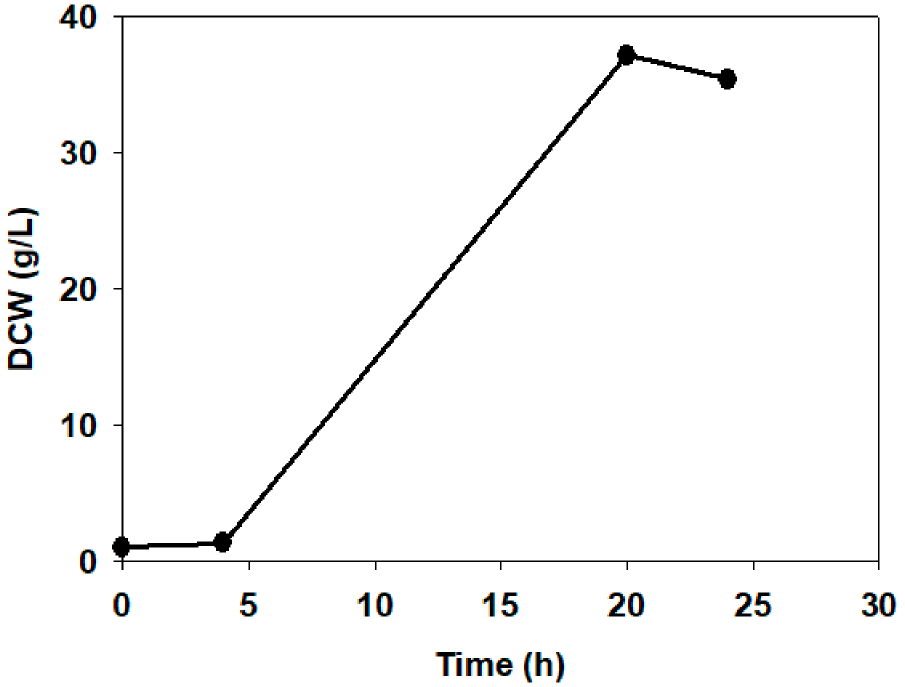

Supplement: Supplementary file 1 [file microorganisms-13-00022-s001.zip › microorganisms-3381008-supplementary.pdf]
